# A qualitative study of governance of evolving response to non-communicable diseases in low-and middle- income countries: current status, risks and options

**DOI:** 10.1186/1471-2458-12-877

**Published:** 2012-10-16

**Authors:** Manju Rani, Sharmin Nusrat, Laura H Hawken

**Affiliations:** 1 Senior Technical Officer (Health Research Policy), Western Pacific Regional Office, World Health Organization, Manila, Philippines; 2 Princess Alexandra Hospital, 199 Ipswich Road, Brisbane, Queensland, 4102, Australia; 3 Technical officer (Health Services Development), Western Pacific Regional Office, World Health Organization, Manila, Philippines

**Keywords:** Cambodia, Fiji, Malaysia, Mongolia, Philippines, Non-communicable diseases, Governance, Policies

## Abstract

**Background:**

Segmented service delivery with consequent inefficiencies in health systems was one of the main concerns raised during scaling up of disease-specific programs in the last two decades. The organized response to NCD is in infancy in most LMICs with little evidence on how the response is evolving in terms of institutional arrangements and policy development processes.

**Methods:**

Drawing on qualitative review of policy and program documents from five LMICs and data from global key-informant surveys conducted in 2004 and 2010, we examine current status of governance of response to NCDs at national level along three dimensions— institutional arrangements for stewardship and program management and implementation; policies/plans; and multisectoral coordination and partnerships.

**Results:**

Several positive trends were noted in the organization and governance of response to NCDs: shift from specific NCD-based programs to integrated NCD programs, increasing inclusion of NCDs in sector-wide health plans, and establishment of high-level multisectoral coordination mechanisms.

Several areas of concern were identified. The evolving NCD-specific institutional structures are being treated as ‘program management and implementation’ entities rather than as lead ‘technical advisory’ bodies, with unclear division of roles and responsibilities between NCD-specific and sector-wide structures. NCD-specific and sector-wide plans are poorly aligned and lack prioritization, costing, and appropriate targets. Finally, the effectiveness of existing multisectoral coordination mechanisms remains questionable.

**Conclusions:**

The ‘technical functions’ and ‘implementation and management functions’ should be clearly separated between NCD-specific units and sector-wide institutional structures to avoid duplicative segmented service delivery systems. Institutional capacity building efforts for NCDs should target both NCD-specific units (for building technical and analytical capacity) and sector-wide organizational units (for building program management and implementation capacity) in MOH.

The sector-wide health plans should reflect NCDs in proportion to their public health importance. NCD specific plans should be developed in close consultation with sector-wide health- and non-health stakeholders. These plans should expand on the directions provided by sector-wide health plans specifying strategically prioritized, fully costed activities, and realistic quantifiable targets for NCD control linked with sector-wide expenditure framework. Multisectoral coordination mechanisms need to be strengthened with optimal decision-making powers and resource commitment and monitoring of their outputs.

## Background

The burden of chronic non-communicable diseases (NCDs) is growing in low- and middle-income countries (LMICs) alongside persistent communicable diseases, poor maternal health, and fragile health systems. NCDs such as cardiovascular disease (CVD), cancers, diabetes, and chronic obstructive respiratory diseases are increasingly affecting people in the economically productive age- groups. NCDs also account for more than 50% of total premature mortality (i.e. deaths in those under 60 years of age) in most LMICs [1,2]. The associated costs can overwhelm households, health systems and national economies.

Encouragingly, NCDs are now high on the global agenda with a series of high-level events, such as the Health Ministers' meeting in Moscow and a United Nations High-Level Ministerial summit, held in September 2011. Calls are being made to establish a Global Fund for NCDs and a global facility for NCD medicines [3]. Past experience of scaling up efforts to tackle specific diseases offers important lessons, especially from the perspective of governance, which should inform the new global efforts for NCDs. The institutional arrangements put in place to plan, manage, coordinate and monitor initiatives aimed at specific diseases such as HIV/AIDS, Tuberculosis and Malaria during scaling up led to ‘segmented service delivery’ which affected effectiveness and efficiency of response to not only the specific initiative itself but also the overall health systems [4-7]. Specific recommendations on governance of the NCD response are already emerging, such as the establishment of a national commission for NCDs [8] and the need for a programmatic 'public health approach' within the context of overall health systems [9].

The organized response to NCD is still in infancy in most LMICs. There is limited published evidence on how this response is structured from governance perspective within the context of overall health systems. We examine the evolving response in terms of institutional structures and arrangements, policy content and development processes, and coordination across different actors and sectors.

Although our research is focused on LMICs in the Western Pacific Region (WPR) of the World Health Organization (WHO), we hope the findings may be of wider interest.

## Conceptual framework

**Figure 1:**
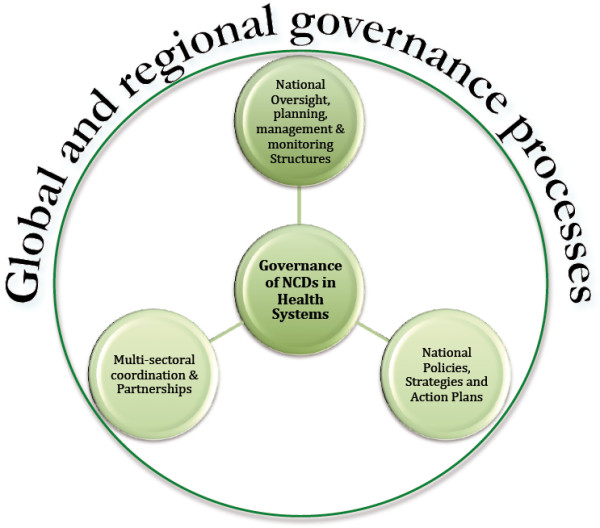
The conceptual framework to analyze the governance of response to NCDs.

Governance and leadership is a central building block for health systems [10]. The term governance is used widely in public administration, with little agreement on the definition. In this paper we use the term to refer collectively to institutional arrangements and management processes that include the setting of overall directions through policy development and coordination mechanisms aimed at delivering an acceptable range of outcomes. The architecture of governance may influence the efficiency and effectiveness of a health system's activities by ensuring best use of resources and reducing duplication and redundancy in the system. This paper focuses mainly on national-level governance mechanisms for responding to NCDs, as these are critical in providing stewardship and mobilizing the necessary political commitments, and may also define the subnational and service delivery arrangements. We conceptualize the governance systems along three key dimensions (Figure 1).

*Governance structures*: The roles and responsibilities, inter-relationships, and architecture of the institutional structures within Ministries of Health (MOH) that are involved in oversight, management, and planning for NCDs were examined. We conceptualize the institutional structures in two categories: NCD –specific structures whose remit is confined to NCDs only, and the ‘sector-wide’ structures responsible for ‘shared’ health functions across different diseases and programs (e.g. human resource development, health planning, information, etc.).

*Policy development and planning*: Policies and plans may be NCD-specific and ‘sector-wide’ (i.e. national health plans) covering all the programs and diseases and other sector-wide issues (e.g. human resources, health financing, information, etc.). We examine the extent to which the content, and processes for development of NCD-specific and sector-wide health policies and plans are aligned.

*Multisectoral coordination, building coalitions and partnerships:* Effective regulation of and influence over the life-style and other environmental determinants of NCDs require interventions across multiple sectors and stakeholders increasing the salience of multi-sectoral coordination. We examine the status, nature, roles and responsibilities, and outcomes of multisectoral coordination mechanisms, coalitions and partnerships both within and outside government.

In addition, the conceptual framework envisages that the governance of health systems and disease-specific programmes, especially in aid-dependent countries, are influenced by architecture of funding flows, policies, accountability and reporting requirements at global and regional level [11].

## Methods

A qualitative desk-review of policy and programme documents (published between 2000 and 2011 and available in public domain) covering the health sector and NCDs was conducted for the five selected countries—Cambodia, Fiji, Malaysia, Mongolia and the Philippines. These countries represent a range of contexts from low-income (Cambodia and Mongolia) to lower-middle income (the Philippines) to upper middle income countries (Fiji and Malaysia). A specific focus of analysis was the most recent national NCD plan and sector-wide national health plan in each of these five countries [12-22]. In addition, policies, strategies, and other information published on the websites of Ministries of Health from other countries in the WPR were reviewed on specific issues.

The paper also draws on data collected in 2004 and 2010 from member states in the WPR as part of the WHO sponsored global key-informant surveys. The surveys assessed the capacity of member states to respond to NCDs in five areas: public health infrastructure for NCDs; the status of policies, strategies, action plans and programmes relevant to NCDs; health information systems, surveillance and surveys; the capacity of health care systems for early detection, treatment and care of NCDs; and health promotion, partnerships and collaboration [23,24]. Both the surveys in 2004 and 2010 used structured questionnaires which were sent to the Ministries of Health. 35 out of 37 countries and areas in the Region participated and returned the completed questionnaire in 2010. The respondents were national NCD focal points. The data from both surveys are publicly available through the Global Health Observatory of WHO [25]

This paper also uses other peer-reviewed literature on governance of health systems and NCDs published since 2000 with special reference to Western Pacific Region.

## Results

### Governance of NCDs: the current situation

#### Governance structures

An increasing number of countries have established special units or departments specifically for NCDs, giving a clear institutional identity to control and prevention of NCDs within MOHs. Globally, countries reporting such structures increased from 70% to 95% between 2000 and 2010 [23]. In the WPR in 2010, all 18 LMICs and 10 out of 12 high-income countries (HICs) reported such structures compared to only 10 LMICs and 4 HICs in 2004. Similar progress has been observed in the WHO European and South-east Asian regions [24,26].

The location and remit of NCD units within the MOH varies across countries (Table 1). In Malaysia and the Philippines, the NCD unit sits alongside communicable disease control units under an overall organizational structure for disease control and prevention. In Cambodia, Department of Preventive medicine is responsible for NCD control and prevention and is separate from the Department of Communicable Disease Control. In Fiji, the Director of Public Health oversees the NCD advisor along with advisors for health promotion, environmental health and nutrition. In Mongolia, the MOH has mainly planning and policy setting functions and delegates the implementation of the programs to its various implementing agencies: the Department of Public Health Policy Implementation and Coordination in MOH in Mongolia has an Officer-in-Charge (OIC) of NCD prevention along with OIC for nutrition & food safety, environmental health, chemical safety, and communicable disease control. The Officer-in-Charge coordinates the implementation of NCD programs through specific implementation agencies such as the National Cancer Centre and the Government Implementing Agency—Department of Health.

**Table 1:** NCD-specific national governance structures in selected countries in WPR, 2010

**Country**	**Location of NCD unit**^**1**^	**Total staffing**^**2**^**(2010)**	**Year of creation**^**2**^	**Reported role and responsibilities**^**2**^
Cambodia	Department of Preventive Medicine	4 full time, 2 part-time	1998	Planning, coordination of implementation, policy development, Monitoring and evaluation (M&E)
Fiji	NCD advisor under Director, Public health	2	2004	Policy development, planning, M&E, implementation
Malaysia	Separate section in overall disease control division	11	1996	Planning, coordination of implementation, M&E
Mongolia	*Officer-in-Charge* of NCD prevention policy implementation and coordination in the Department of Public Health Policy Implementation and Coordination in MOH	4	1997	Planning, coordination of implementation, M&E
Philippines	Degenerative disease division of National Center for Disease control and Prevention	13	1998	Policy development, coordination of implementation, M&E.

*Source:*^*1*^*qualitative review;*^*2*^*data from WHO Key-informant global survey (2010).*

##### An integrated NCD technical team or organizational structures by specific diseases or risk factors?

Co-existence of different NCDs in a substantial number of patients, and the common risk factors that link major NCDs (i.e. tobacco use, unhealthy diets, physical inactivity and harmful use of alcohol) warrant integrated multidisciplinary approach to disease prevention and management. Integrated approaches can improve efficiency, which is especially important given the often limited human resources in LMICs. Yet disease- or risk-factor based governance structures, often separate and additional to the overall NCD specific structures described above, have developed over time in some countries. For example in Vietnam, the National Institute for Cardiovascular Disease manages the program on prevention and control of CVD; the National Institute of Oncology (National Cancer Hospital) manages the program on prevention and control of cancer; the National Institute of Endocrinology and the National Institute of Psychiatry each manage the separate national programs on diabetes and mental illness, respectively [27]. A similar situation is seen in Mongolia, with the National Cancer Center of Mongolia responsible for the policy development, implementation and management of the National Cancer Control Program. In the Philippines, the NCD program, started in 1986, operated vertically from national to the local level as three parallel disease-specific programs including the Philippine Cancer Control Program, the National CVD Prevention and Control Program, and the National Diabetes Mellitus Prevention and Control Program [20]. The 1996 National Diabetes Act in the Philippines also created the National Diabetes Commission to coordinate the overall prevention and control of diabetes mellitus [28].

Disease- or risk-factor specific global or regional initiatives seem to have also influenced development of disease- or risk-factor specific organizational structures within MOHs. For example, much stronger global advocacy and funding flow for tobacco control under the WHO Tobacco Free Initiative has led to far more resourced tobacco control teams in LMICs, often separate and under different hierarchical control from the NCD teams described above. Encouragingly, some positive trends are observed in establishment of more integrated NCD programs. The Philippines merged the three vertical programs into an integrated NCD control and prevention program in 2000, and similar trends are seen in other countries such as Mongolia and Malaysia.

##### Inter-relationships among multiple organizational structures with NCD related functions and with structures responsible for sector-wide health system functions

The global key informant survey in 2010 elicited information about the planning, coordination of implementation, and monitoring and evaluation of NCD initiatives and almost all the member states in the WPR reported NCD-specific units responsible for these functions. However, the relationships and division of roles and responsibilities between NCD units and other sector-wide institutional structures, such as policy and planning units and health information units, are often not clearly specified. In some countries, the NCD related structures seem to function mainly as technical units and not as specific programme management and implementation entities. For example, in contrast to the situation in Vietnam described earlier, structures such as the National Cancer Centre, National Cardiovascular Centre, National Centre for Neurosciences and Psychiatry affiliated with Ministry of Health, Labour and Welfare in Japan and in many other developed countries including Malaysia mainly serve as leading technical and research institutions in their respective fields, rather than as programme management or operational entities [29].

Many LMICs have other major organizational structures with NCD related functions such as health promotion centres and national centres for nutrition, and these are often distinct entities under different hierarchical control. For example, Cambodia and the Philippines have an autonomous National Centre for Health Promotion. In Mongolia the National Centre for Health Development has the key responsibility for health promotion. Many countries including Malaysia, Mongolia, and the Philippines have newly established, or plan to establish, health promotion boards or foundations to provide population-based primary prevention services for NCDs. However, relationships of these newly proposed structures with the existing organization structures for health promotion or the NCD units are not clearly specified. There is a potential overlap in the reported functions and responsibilities, as NCD units also reported health promotion as their key responsibility in the global key informant survey. For example, the National Centre for Health Promotion in Cambodia (distinct from Department of Preventive Medicine which is designated organizational structure for NCD control) is the focal organization for tobacco and alcohol control and is mandated to lead on matters of behaviour change, working across multiple programmes [30]. This sort of overlapping infrastructure for health promotion raises concerns about lack of co-ordination and potential duplication, a problem previously experienced in Cambodia with programmes such as maternal and child health, which developed their own behaviour change programmes and resources not necessarily involving the National Centres for Health Promotion [30].

If the common goals and targets articulated in sector-wide and NCD-specific policies and plans are to be pursued effectively and efficiently, clear specification of roles and responsibilities of each institutional structure involved may be required to align the NCD-related functions, activities and resources located in multiple and distinct organizational structures across the MOH.

Neither the global key informant survey nor the qualitative review indicated the presence of formal institutional coordination mechanisms for these different organizational units within the MOH. The Philippines has a Sectoral Management and Coordination Team responsible for the overall development, monitoring and coordination of policies, mechanisms and guidelines for the health sector, but its role in coordinating NCD policies across the Department of Health is not known.

Cambodia provides a ‘typical’ example of the situation witnessed in many countries on how additional “vertical” disease- or program-specific structures were created over time that evolved into self-contained units taking over sector-wide health system functions relative to only that particular disease/program leading to segmented service delivery.

**Figure 2:**
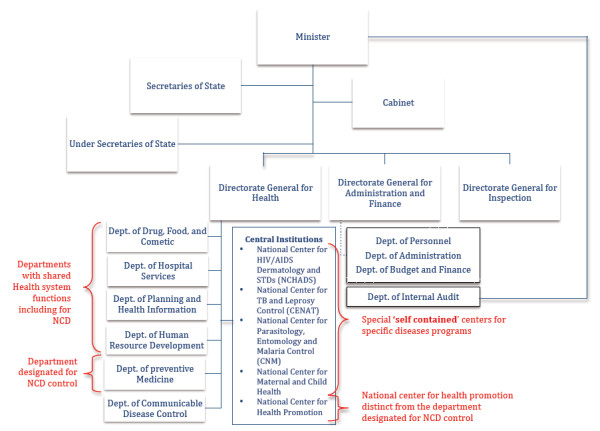
**Diseases-specific structures and overarching health functions structures in Ministry of Health, Cambodia. ***Adapted from official organizational chart provided in the MOH website. The figure shows only the departments and centers relevant to the discussion in the paper and not all the department and centers in MOH. *

In the past, the MOH in Cambodia created special institutions such as the National Centre for HIV/AIDS and STDs, the National Centre for TB and Leprosy Control, the National Centre for Parasitology, Entomology and Malaria Control, and the National Centre for Maternal and Child Health (NCMCH), which respectively became responsible for HIV/AIDS, Tuberculosis, Malaria and immunization services (Figure 2). These national centers were predominantly funded by distinct external donors. Over time, these centres expanded with additional staff to become self-contained ‘management and implementation units’ also managing 'horizontal' sector-wide functions, such as surveillance, financing, human resource development, medical supply and logistics, and service delivery, but for that particular disease/program only. This led to duplication of many functions with other departments including the Department of Communicable Disease Control, the Department of Planning and Health Information, and the Department of Human Resources.

Currently, the Department of Preventive Medicine is responsible for NCD control and is expected to facilitate and coordinate the development of NCD policy, conduct surveillance of NCD risk factors, develop treatment guidelines and deliver health promotion, prevention and treatment services and training relevant to NCD prevention and control. Many of the reported functions of this NCD unit overlap with other sector-wide centres/departments that are expected to perform some of these functions across different programmes/services (Figure 2).

If the experience of other disease-specific structures mentioned above is repeated, these NCD-specific structures may start growing by having additional staff with expertise and responsibility in the areas such as planning, management, human resource training, supervision, medical supply, logistics and procurement, and health information systems to become self-contained programme implementation and management entities undermining overall consolidation of these sector-wide functions in MOH. Encouragingly, Cambodia sector-wide health plan recognizes the fragmentation of activities, funding, monitoring and supervision and administrative lines of authority [13]. The NCD-specific plan also acknowledges other organizational structures related to NCD-control and the need to coordinate with departments with sector-wide functions [12]. However, suggested solutions in these plans in the form of more ‘coordination’ and ‘integration’ remain vague and the issue of clear specification of roles and responsibilities has not been addressed.

#### Policy development and planning

In LMICs in the WPR, the sector-wide national health plans and policies increasingly mention NCDs and included at least one goal or objective related to NCD control, although the level of detail varies. In Malaysia, NCD policy agenda figures high up not only in the health sector plan ([16] but also in the Tenth Malaysia Plan (2011–2015), a blue print for overall national development prepared by the national Economic Planning Unit [31]. However, in general in most of the countries examined, NCDs appear less often and less prominently in other sector-wide plans such as national human resource policies and national finance policies.

An increasing number of countries reported development of disease- and/or risk- factor specific as well as integrated NCD strategies or plans. By 2010, 80% of countries and areas in the WPR, compared to 41% in 2004, reported NCD-specific national policies/plans in the global key informant survey. Similarly, in the WHO European Region the proportion of countries having a national NCD specific policy rose from 57% in 2000–01 to 83% in 2005-06 [24]. This substantial increase in the number of countries developing NCD-specific policies after 2000 may partly reflect the influence of the global strategy on NCDs endorsed by the World Health Assembly in 2000 which led to increased in-country technical assistance and dialogue by international partners for developing such NCD-specific policies [17,32].

In addition to the integrated plans for NCDs control, many countries in the WPR reported having multi-year plans for specific NCDs (e.g. diabetes mellitus, CVD) or for a specific risk factor such as alcohol or tobacco use, unhealthy diet, obesity, and physical inactivity. In 2010, the highest number of countries reported a policy/strategy for diabetes mellitus (71%) and cancer control (66%) and the lowest number reported for chronic obstructive pulmonary diseases (11%). These developments do not necessarily reflect the relative public health importance of these diseases. For example, the estimated number of deaths from cardiovascular (4.1 million) and chronic respiratory diseases (1.5 million) is much higher than that of diabetes ( 0.2 million) in the WPR [2]. For risk factors, the highest number of countries reported having a strategy for tobacco control (83%) followed by healthy diet (66%) and the lowest number for alcohol use (54%). Similar patterns were seen in the WHO European and South-East Asia Region [24,26]. The chronology of global or regional issue-specific initiatives seems to be correlated with these patterns of disease- or risk-factor specific policies and availability of budget especially in LMICs. For example, reports of an issue-specific strategy on diabetes and tobacco control by a large number of LMICs in the WPR probably reflects the influence of the Western Pacific Declaration on Diabetes in 2000 and the WHO framework convention for tobacco control in 2005, respectively. Fewer high profile regional/global initiatives for chronic obstructive respiratory diseases or alcohol may in part explain why fewer countries reported a specific strategy/program on these issues.

A qualitative review of sector-wide and NCD-specific polices/plans in five countries showed weak alignment between the two groups of policies in terms of goals and targets, financial resource allocation, and implementation mechanisms, potentially reflecting the largely autonomous development process of these policies involving different constituents. The NCD-specific Plans do not necessarily expand on the directions set in sector-wide national health plan and may be completely independent. The development of NCD-specific plans are for the most part coordinated by the NCD unit in the MOH, the role and influence of the sector-wide policy and planning units in the development of these policies is not very clear.

An important factor in ensuring the implementation of NCD-policies and plans is the extent to which political consensus and administrative undertakings to ensure implementation are in place. This might best be achieved by involving key stakeholders at the preparatory stages of NCD strategies but the extent to which this happens is not clear from our qualitative review and practice seems to vary. The 2007 National NCD control program document in Mongolia identifies all the policy actors and their roles, and was explicitly endorsed by the Ministry of Food and Agriculture, Ministry of Finance, and governors of the capital city and provinces ([19]. However, the national NCD plans for Cambodia, Malaysia and the Philippines identify the key policy actors and their responsibilities, but give no information on whether policy roles and responsibilities have been discussed with them, agreed to and endorsed by them [12,17,20]. Fiji's policy describes the consultation process, which is limited mainly to health actors and international agencies/partners [14].

Of the five countries examined, only Fiji NCD Plan included any consideration of the costing and financing of proposed activities for NCD control and prevention ([14]. None of the plans (either sector-wide or NCD-specific) provided any analysis on the current level of public health expenditures (PHE) for NCDs (Table 2), despite several studies that have shown a substantial proportion of PHE being spent on treatment and care of NCDs [33]. Fiji's NCD plan indicated a total annual budget of US$ 226,199 or US$ 0.27 per capita ([14], much less than the US$ 1.0-1.5 estimated just for tobacco control and salt reduction efforts based on only information, education and communication, and regulatory measures in LMIC [34].

**Table 2:** Status of costing/financing details for NCD prevention and control in NCD-specific and sector-wide national health policies/plan

**Country**	**Sector-wide health plan/policy**	**NCD specific plan/policy**	**Baseline expenditure levels given**	**Sources of financing defined**	**Potential gaps in financing identified**
Cambodia [12,13]	Costing and financing of reproductive health and child survival interventions but not NCD ([13]	No costing/budget for the proposed activities provided.	Overall public spending on health accounted for 12% of national budget, 1% GDP	Proposes an increase in government budget, no specific financing sources for NCDs.	Identifies need to mobilize additional resources for NCD, health promotion, traffic injuries, but with no quantification ([13]
Fiji [14,15]	No costing provided [15]	Annual budget provided: $226199 $0.27 per capital [14]	For overall health systems (2.87% of GDP for MoH), but no NCD-specific budget [15]	Annual Increase to health budget by 0.5% for 5-7 yrs; no specific financing for NCDs [15]	Establish Health Care Financing Unit to identify gaps in the system, not specifically for NCDs [15]
Malaysia [16,17]	No costing/Budget provided	No costing/ budget provided	No	No	No
Mongolia [18,19]	Medium-term expenditure framework, but no clear costing linked to proposed activities[18]	No costing/ budget provided [19]	For overall health systems, but no NCD-specific[18]	No	notices additional resources need to implement the Health Sector Strategic Master Plan [18]
Philippines [20,21]	Allocates budget for Health Promoting activities for 2006–2007 [21]	No costing/ budget provided, asks the local government units to establish effective financing schemes at provincial and local level [[20]	Not given	Sources of funding identified[21]	Identifies a gap of 11% of the required costs for overall health systems (PhP3.9 Billion) [21].

An important element of a modern health policy approach is the setting and monitoring of quantifiable goal and targets*,* to generate a sense of political urgency and serve as a basis to hold various stakeholders accountable. While both the sector-wide and NCD-specific plans in all the five countries included quantitative targets related to NCD control, substantial discrepancies were observed in the targets proposed between the two group of policies (Table 3). Few plans presented the baseline levels or measurement process for the proposed indicators, and almost no country commented on the past trends for the selected indicators despite documentation of implementation of several NCD related programmes in the past (Table 3). The only baseline data presented in Fiji's NCD plan (2010–2014) comes from 2002, despite implementation of the national NCD plan 2004–2008 in between. In the majority of countries examined, the quantified targets/goals were unclear, difficult to measure and in many cases inappropriate. For example, many countries set targets for reducing the prevalence of diabetes or hypertension (Table 3), which are almost impossible to achieve due to the likelihood of improved diagnosis and more people surviving with chronic disease as a result of better treatment. While Fiji's National NCD Plan sets a goal of reducing the overall prevalence of diabetes by 5% in four years that may be epidemiologically unrealistic, Malaysia has set a more achievable target of halving the rate of increase in diabetes prevalence from 0.4% per year to 0.2% per year, rather than reducing the overall prevalence per say.

**Table 3:** Selected indicators and targets for NCD prevention and control in national health policies/plan and in NCD specific plans: Poor alignment and poor selection of indicators

**Country**	**Sector-wide health plan/Policy**	**NCD-specific plan/policy**	**Baseline levels**
**Cambodia**	Reduce between baseline (2005-08) and 2015,	Sample indicators to monitor progress mentioned, but no targets specified.	■ Average baseline given for 2005-2008 ([13]; data from existing literature summarized in NCD-specific plan
	■ the adults smoking prevalence% (male/female) from 54/9 to 44/2		■ Proposes monitoring through national STEP surveys
	■ Incidence of hypertension per 1000 population from 20 to 15		
	■ Prevalence of adults with diabetes reported from public facilities from 2 to <2		
	■ Incidence of cervical cancer per 10,000 population reported from public facilities from 25 to 12.5 [13]		
**Fiji [**[14]**,**[15]**]**	By 2015, reduce prevalence of	By 2014, reduce prevalence of	■ Baseline from National NCD STEPS Survey 2002, no progress reported from prior National NCD Strategic Plan 2004-2008
	■ Diabetes (25-64 yr) from 16% to 14%	■ Diabetes by 5%	■ Proposes monitoring through National NCD STEPS Survey and National Nutrition Survey.
	■ Alcohol related injuries to less than 5%	■ Common risk factors by 5%	■ No periodicity/monitoring agency defined.
	■ moderate physical activity by 5%	■ Intermediate risk factors by 5%	
	■ fruing/vegetable intake (Adults) by 5%	■ Major NCDs by 5%	
	■ Current smoking (15-65yrs) from 37% to 33%	■ Tobacco use: 10% from baseline	
	■ reduce obesity by 6.2%	■ Improve nutrition: No target	
	■ Increase HPV vaccine coverage by 5%	■ Alcohol related harm: No target	
		■ Cardiovascular diseases by 5%	
		■ Improve national NCD status by 5%	
**Malaysia [**[16]**,**[17]**]**	■ Indicators are listed as prevalence of Ischemic heart disease, mental illness, CVD, Diabetes, cancer and chronic obstructive respiratory disease but with no specific targets.	By 2016, Reduce Prevalence of	■ Baseline Data from National Health and Morbidity Survey 2006
		■ Diabetes from 11.6 to <13.6%	■ Proposes monitoring through Behavioural Surveillance Survey, NCD Risk Factor Surveillance
		■ Obesity from 26.2% to <33.7%	■ Periodicity/Monitoring agencies identified
		■ Healthy Eating – no target given	■ No past progress reported
		■ Physical Activity – no target given	
**Mongolia**[18,19]	Between 2010 and 2015, Reduce prevalence of	Between 2009 and 2013, Reduce prevalence of	■ Some baseline data given from 2004-2005
	■ Daily Smoking from 37% to 31%	■ Smoking from 23.4% to 20.4%	■ Mechanism to collect data and its periodicity not defined
	■ Daily salt intake (gm/day) from 13g to 12g	■ Daily salt intake (gm/day) from 9.6 to 9.1	■ No achievements or rate of progress described in the immediate past to inform the current target setting
	■ Increase the percentage of adult population that reduce alcohol intake to 2-3 std /wk from 30% to 40%	■ Alcohol use among population (last month) from 29% to 27%	
	■ Increase population doing fitness activities at least 3 times/wk from 20% to 25%	■ Increase in population with active life-style on regular basis with minimum of 30 minutes from 18.4% to 23.4%	
**Philippines**[20,22]	Between 2006 & 2010, reduce prevalence% of	This is an operation manual. Indicators to be monitored are outlined but no quantified national targets for these indicators are given	Some baseline data 2000/2003
	■ Obesity from 4.3 to 3		Proposes Behavioural Risk Factor Surveillance System including Adult
	■ Smoking from 34.8 to <34.8		National Nutrition and Health Survey to monitor the progress.
	■ Alcohol from 46 to <46		No reporting of past progress
	■ Inactivity from 60.5% to 50.8%		
	By 2010, reduce mortality rates (per 100 000) to less than the baseline level in 2006 for		
	■ CVD < 79.1		
	■ COPD<63.2		
	■ Diabetes <20.8		
	■ Cancer <47.7 [22]		

Notes: The indicators are listed exactly in the same way as written in the respective plan/policy, however, for some countries, only selected indictors/targets and not all the indicators/targets given in a plan, are listed here for the sake of brevity.

#### Multisectoral coordination, building coalitions and partnerships

In 2010, 32 out of 35 member states in the WPR reported partnerships in implementing NCD activities in the global key informant survey. The reported key stakeholders for partnerships included non-governmental organizations (86%), other non-health government ministries (83%), private sector (71%), other international organizations (66%), academe (60%) and UN agencies (51%).

##### Multisectoral coordination

The most commonly reported mechanism in the global key informant survey for multisectoral coordination was cross-departmental or inter-ministerial committees (80%). However, the survey does not give details of the composition, roles, responsibilities, and effectiveness of these partnerships, or the extent to which these partnerships influence national strategies, plans and regulations for NCDs.

Encouragingly, in all the five countries, both the sector-wide and the NCD specific plans emphasize the importance of multi-sectoral coordination and identify relevant stakeholders. The qualitative review showed that high-level multisectoral coordination committees exist in all these countries (Table 4). However, in Cambodia, the mandate of current interministerial committee is limited to tobacco control only, though the NCD-plan proposed to establish an inter-ministerial working group by 2009, which was also expected to help with the next NCD plan [12].

**Table 4:** Intersectoral coordination mechanisms in selected countries in WPR, 2011

**Country**	**Name of intersectoral mechanism**	**Chair**	**Membership**	**Year of establishment**	**Comments**
Cambodia	Inter-ministerial committee for education and reduction of tobacco use;	Minister of Health	12 government ministries and institutions	June 2001	plays a major role in
					formulating the National Strategic Plan on tobacco control, law and legislation fortobacco control.
					NCD plan mentions about establishment of inter-ministerial working group by 2009, but status is not known at the time of study.
Fiji	National NCD committee (similar multisectoral committee for health promotion, HIV/AIDS and suicide prevention.	Minister of Health	permanent secretary or directorate level of government, non-state actors and civil society groups, including faith-based groups	2004	Coordinate national implementation of the respective strategic plans developed by the same multi-stakeholders.
Mongolia	National Council for public health	Prime-minister	Minister-level member ship from 8 line ministries (health, education, justice, infrastructure, food and agriculture, environment, foreign affairs and defence), the National Statistical Office, the HSUM and the Ulaanbaatar City Government	2002	Another multi-sectoral structure is Health Promotion Foundation headed by Minister of Health with membership from director, taxation office, Ministry of Finance.
Malaysia	Cabinet Committee for a health promoting environment proposed in the National Strategic plan for NCD (2010–2014)	Deputy Prime-minster	Minister-level membership from 10 line ministries	2011	Has clear terms of reference to determine policies that support positive behavioural changes towards healthy eating and living. The Committee held its first meeting in April 2011.
Philippines	Philippine coalition for prevention and control of NCD It institutionalized the annual public health forum on NCD prevention and control since 2006.	NA	Initial membership has 44 organizations including various medical specialty organizations and societies, professional organizations, non-government organizations, government agencies, academe.	2004	Each member organization signs an Memorandum of understanding that it will contribute to the programs and activities approved by the Coalition Council in consonance with its mandate, while maintaining its own independent programs and avoid open conflict with similar actions of the Coalition.

The roles and responsibilities, powers and legitimacy of decision-making, and resources of these coordination mechanisms were often unclear. Evidence is already emerging that suggests the limited functionality and effectiveness of these structures in influencing either policies or resource allocation in non-health sectors. For example, although never formally abolished, the national public health council chaired by Mongolia's Prime Minister is not active [35]. A similar situation has been reported from Lithuania, outside the WPR, where an intersectoral committee with vice ministers from a number of ministries was established in 2002 and reported to be not very active [36].

##### Partnerships with non-state actors

Effective governance for NCD at national level requires the development of effective partnerships and coalitions to generate the demand for change and to catalyze political action. The range of actors and stakeholders for non-communicable disease control are complex and include food manufacturers and retailers, tobacco and alcohol industries, civil associations, disease/condition specific advocacy groups such as national diabetic associations, and professional associations. Although the multisectoral coordination mechanisms in both Fiji and the Philippines included membership of non-governmental organizations (NGOs) (Table 4), LMICs may not be well equipped to develop partnerships or engage effectively with such a complex wide-range of actors, often with conflicting interests. In the global key informant survey, almost 86% of member states in the WPR reported partnerships with NGOs, and 71% with private industry. However, functional mechanisms to deal with non-traditional stakeholders, such as food manufacturers, do not seem to be well established in any of these countries.

##### Partnerships and coordination with international donors and technical partners

While, the implementation of programs for communicable diseases and maternal and child health in LMICs owe much to the financial and technical assistance of external donors, less than 3% of global development assistance for health currently goes to NCDs [37]. Despite mounting international advocacy, the future of the international financing for NCDs is still unpredictable.

In the past, many global initiatives had required countries to establish coordination structures for specific health issues as a condition to receive the assistance. For example, the Global Fund required a Country Coordination Mechanism and GAVI Alliance required an Interagency Coordination Committee (ICC), often having similar membership, functions and mandates [38]. Similarly, development banks (e.g. Asian Development Bank, World Bank) have promoted the creation of special Project Management Units. These coordination mechanisms focused on monitoring and information sharing about implementation of specific donor funded activities, rather than a genuine engagement with all the external partners to harmonize their efforts across them, in-line with national priorities [38]. No such NCD-specific coordination structures targeted to external donors/ technical partners currently exist, although in the global key informant survey 51% of countries in the WPR reported partnerships with UN agencies, and 66% with other international organizations.

## Conclusions

The analysis in the paper is limited by explicit information available in the public domain and may not be able to capture all the complex consultations or discussions that may be ongoing in the countries more recently. Hence the results should be interpreted in light of this limitation.

The evolving response to NCDs in LMICs shows several positive trends. These include increasing institutional recognition of NCDs, a move from disease-based programs to integrated NCD programs, and an increasing inclusion of NCDs in sector-wide health plans. These developments reflect the increasing recognition of the high burden of NCDs, and an explicit acknowledgment of the need for multisectoral actions with the creation of high-level coordination mechanisms. The analysis also suggests substantial influence of supranational initiatives and processes on in-country governance structures and policy development processes, which offers both opportunities and challenges.

Notwithstanding the above positive trends, the analysis highlights some areas of concern. Each of these areas of concern and potential strategies to address them are described below.

### Institutional arrangements for responding to NCDs

The current directions in development of NCD-specific institutional structures in LMICs with their increasing direct role in planning, management and implementation may lead to segmented service delivery systems for NCDs, especially with increase in NCD-specific external funding, as experienced in the past with scaling-up of programs for immunization, malaria, Tuberculosis, HIV/AIDS.

Two key strategies are recommended to pre-empt this undesirable outcome. The first strategy would be clear separation of the ‘technical’ and the ‘operational and programme management’ functions between NCD-specific units and sector-wide organizational units (e.g. health policy & planning units, human resource development units, drug and logistics units), respectively. The NCD-specific structures are justified to build much needed technical capacity for NCDs in MOH and to provide institutional identity and visibility, especially when similar structures exist within the MOH for other public health problems perceived to be important. However, it will be critical that these NCD-specific units are developed only as technical advisory bodies with strengthening of their capacity in analyzing up-to-date technical information, development of clinical guidelines, advising on suitability of different proposed interventions and research. In addition, NCD-specific units should focus on strategizing, guiding, coordinating policies and activities across different stakeholders within and beyond MOH. However, these units should not act as direct implementing bodies for NCD plans and management and delivery of NCD-related services which should be rather left to sector-wide organizational structures.

Second, the resources to strengthen institutional capacity to respond to NCDs should not be solely targeted to NCD-specific units, but more importantly to sector-wide organizational units to build their capacity in effectively incorporating NCD-specific requirements in human resources development, health financing, medical supply and logistic and information systems.

### Visibility and articulation of NCDs in sector-wide health policies and plans

Three major concerns were identified in the increasingly complex NCD policy landscape in LMICs.

First, out analysis suggest that the sector-wide health plans were not entirely informed by critical analysis of local disease burden and health needs. Often local evidence, even when acknowledged in situation analysis, was ignored in favour of global priorities and goals. Hence, NCD-specific units should be fully engaged in the sector-wide health policy and planning process to ensure inclusion of NCDs related activities as appropriate to their epidemiology, morbidity/mortality burden in the sector-wide health plans.

Second, our analysis shows weak alignment (Tables 2 and 3) among sector-wide and NCD-specific policies/ plans suggesting relatively autonomous development at different times by different constituents with unclear linkages. Ideally, the NCD-specific plans should offer a higher-level technical detail expanding on the directions given in the sector-wide plans within the sector-wide operational limits in infrastructure, human and financial resources. NCD-specific plans should be developed with participation and endorsement of all the stakeholders within health and non-health sectors with NCD units playing only a coordinating role. The sector-wide national planning bodies should ensure that NCD-specific plans are within the resources offered by health sector.

Third, NCD specific plans with no information on financial and implementation feasibility and no realistic quantifiable targets, as have been observed in some of the countries examined, may become simply technical papers or 'laundry lists' of desirable activities with limited reference value and no follow-up at the government level [36].

### Effectiveness of multisectoral coordination

Although the need for multisectoral coordination is acknowledged by most LMICs, the resources needed to organize and manage such coordination mechanisms appear to be inadequate. The inter-ministerial bodies—the most common mechanism reported—seem to be relatively fragile structures in most LMICs with limited effectiveness in influencing the policies, programs and resources allocation in different sectors [36]. In some countries, such as Mongolia, these structures are already being reported as inactive [35]. The implementation of 'Health in All Policies' has remained a challenge even in developed countries [39], with few non-health ministries taking action on their own to reduce deaths from cancers or hypertension. This implies that MOH will have the added responsibility for proactive negotiations and coordinating efforts to build stronger multisectoral partnerships. Also, it will be more efficient to set-up these coordination mechanisms for multiple issues that require inter-sectoral coordination, rather than for specific issues (e.g. tobacco) as observed in some countries. Finally, as international initiatives, partners and assistance for NCDs may increase in the near future, countries have to be in the driver's seat for creating coordination mechanisms that harmonize efforts of different partners and agencies. Preferably these will be part of an overall existing health sector coordination mechanism, rather than creating specific multiple coordination mechanisms to fulfil requirements of specific donors.

To summarize, attention to evolving governance structures and policy development processes for NCDs is vital. It will aid pre-emptive and corrective action at an early stage for the effective, efficient, and sustainable scaling-up of response to NCDs within a health systems context.

## Competing interests

The authors declare that they have no competing interests.

## Authors’ contributions

MR conceptualized the paper and wrote the first draft and the final version. SN assisted with the qualitative desk reviews of the data and other literature and reviewed various drafts. LH reviewed the draft of the paper and provided comments and inputs towards the final paper. All authors read, commented, and approved the final version of the manuscript.

## Authors’ information

MR – Western Pacific Regional Office, World Health Organization SN—University of Auckland, New Zealand LH-- Western Pacific Regional Office, World Health Organization.

## Disclaimer

The views expressed in this paper are solely the responsibility of the named authors and do not necessarily reflect the decisions or stated policy of the organization they work for.

## Pre-publication history

The pre-publication history for this paper can be accessed here:

http://www.biomedcentral.com/1471-2458/12/877/prepub
